# Impact of Morphology of Conductive Agent and Anode Material on Lithium Storage Properties

**DOI:** 10.1007/s40820-015-0051-7

**Published:** 2015-06-30

**Authors:** Xiaobing Zhang, Ji Ma, Kezheng Chen

**Affiliations:** Lab of Functional and Biomedical Nanomaterials, College of Materials Science and Engineering, Qingdao University of Science and Technology, Qingdao, 266042 People’s Republic of China

**Keywords:** Lithium-ion batteries, Morphology, Conductive agent, Anode material

## Abstract

In this study, the impact of morphology of conductive agent and anode material (Fe_3_O_4_) on lithium storage properties was throughly investigated. Granular and belt-like Fe_3_O_4_ active materials were separately blended with two kinds of conductive agents (i.e., granular acetylene black and multi-walled carbon nanotube) as anodes in lithium-ion batteries (LIBs), respectively. It was found that the morphology of conductive agent is of utmost importance in determining LIBs storage properties. In contrast, not as the way we anticipated, the morphology of anode material merely plays a subordinate role in their electrochemical performances. Further, the morphology-matching principle of electrode materials was discussed so as to render their utilization more rational and effective in LIBs.

## Introduction

Long-lasting and green rechargeable energies are in high demand for solving the dilemma of global environmental pollution and energy shortage [1–3]. Among various renewable batteries, lithium-ion batteries (LIBs) have attracted increasing attention for their applications in portable electronics and electric vehicles in recent years [4–8]. For the requirement of high-performance LIBs, novel anode materials have become one of the research hotspots nowadays. Magnetite (Fe_3_O_4_) is such a potential candidate because of its attractive theoretical capacity of 924 mAh g^−1^, nontoxicity, natural abundance, low cost, and high electronic conductivity (2 × 10^4^ S m^−1^) [9, 10]. Along these lines, many efforts have been devoted to obtaining high-performance Fe_3_O_4_ anode material with various morphologies, including hollow spheres [11, 12], arrays [12], belts [13], rods [14], fibers [15], etc. Although controlling the morphology of anode material may initially seem like a scientific curiosity, its impact goes far beyond esthetic appeal. For example, the porous morphology with higher surface area is much needed because of the intercalation capacities and affinities for lithium ions (Li^+^) to the more exposed holes in the surface, which could then shorten the diffusion length of Li^+^ [16]. Besides, the inner pores also allow the material to effectively buffer the stress induced during the charge–discharge process [17]. Other desirable morphologies involve one-dimensional nanostructures, like nanorods and nanowires, which can offer a small diameter to enhance lithium diffusion and yet still provide a limited surface area to prevent excessive side reactions [18]. All of these morphological features of anode materials play a significant role in determining the discharge characteristics [19], and thus they are essential to apply in high-performance LIBs.

Apart from anode materials, the types and morphologies of conductive agents are other determinants to LIBs storage performances. Generally, granular carbon black [acetylene black (AB)] is seen as a universal conductive agent with high conductivity. In addition, other types and morphologies of carbon species may be more desirable, and several research efforts have been conducted in this regard. Wang et al. [20] enabled Ni(OH)_2_ nanocrystals to grow on graphene sheets for potential energy storage applications. They found that graphene sheets with low oxidation are qualified to impart excellent electrical conductivity to the macroscopic ensemble of the composite materials (without the need of carbon black additives). The highly conducting graphene network allows rapid and effective charge transport between the Ni(OH)_2_ nanoplates in the macroscopic ensemble and the current collector, allowing for fast energy storage and release. Likewise, Liu et al. [21] utilized carbon-coated ZnO nanorod arrays as anode material without the extra addition of conductive agents. They found the coated carbon arrays not only ensure good electrical contact of ZnO with the current collector and enhance the charge transfer/Li^+^ transport, but also effectively alleviate the strains caused by the volume variation of ZnO nanorod cores and prevent the disintegration. Despite the progress achieved to date, the optimization of morphology of conductive agents with anode materials is not sufficiently discussed. And what is more, very little is known about the morphology-matching principle between the used conductive agents and anode materials.

In this study, we utilize Fe_3_O_4_ anode material (with granular and belt-like morphologies) and two kinds of conductive agent [i.e., granular AB and multi-walled carbon nanotube (MWNT)] as prototype systems to investigate their morphology impact on lithium storage performances. The selection criteria of multi-morphological conductive agents and anode materials are also proposed according to our electrochemical measurements and analyses.

## Experimental

### Preparation of Fe_3_O_4_ Anode Material

Analytical ferrous sulfate (FeSO_4_) and sodium carbonate (Na_2_CO_3_) were purchased from Sinopharm Chemical Reagent Co., Ltd. (China), and used as received without further purification. In a typical synthesis, 2 mmol of FeSO_4_ was dissolved into 50 mL of deionized water at room temperature until a homogeneous solution was formed. After that, 1 g of Na_2_CO_3_ powder was added to the solution with continuous magnetic stirring. Then the mixture was transferred into a Teflon-lined stainless-steel autoclave with a capacity of 100 mL for hydrothermal treatment at 160 °C for 20 h (sample 1, marked as S160) and 200 °C for 8 h (sample 2, marked as S200) separately. The as-obtained precipitates were repeatedly washed with deionized water and ethanol, and finally dried at 60 °C for 6 h.

### Characterization

X-ray diffraction (XRD) patterns were recorded on a powder X-ray diffractometer (Rigaku D/max-rA) equipped with a rotating anode and a Cu K_a1_ radiation source (*λ* = 1.5406Å) at a step width of 0.02°. Field emission scanning electron microscope (FE-SEM) images were collected on a field emission scanning electron microscope (JEOL JSM-6700F). Transmission electron microscopy (TEM) images were performed on a JEM-2100 TEM with operating voltage at 200 kV.

### Electrochemical Measurements

The electrochemical measurements were carried out at 25 °C using 2032 coin-type cells with pure lithium metal as the counter and reference electrodes. The working electrode consists of active material (as-synthesized Fe_3_O_4_ products), conductive agents (AB or MWNT), and sodium carboxymethyl cellulose binder (CMC, 800–1200 mPa s, DS 0.7) in a weight ratio of 60: 20: 20, using deionized water as the dispersion medium. The mixture was spread on a Cu foil and dried under vacuum at 120 °C for 8 h. The electrolyte used was 1.0 mol L^−1^ LiPF_6_ in a mixture of ethylene carbonate and dimethyl carbonate (1: 1 by volume). Cell assembly was carried out in an Ar-filled glove box with the concentrations of moisture and oxygen below 1 ppm. The cells were cycled at different current rates of 0.2, 0.5, 1, 2, and 5 C (1 C = 924 mA g^−1^) between 0.01 and 3 V using a LAND battery tester. Electrochemical impedance spectroscopy (EIS) measurements were carried out on a CHI660D electrochemical workstation by applying a sine wave with an amplitude of 10.0 mV over a frequency range of 100 kHz to 10 mHz. The voltages mentioned in this study were referred to a Li/Li^+^ redox couple.

## Results and Discussion

**Fig. 1:**
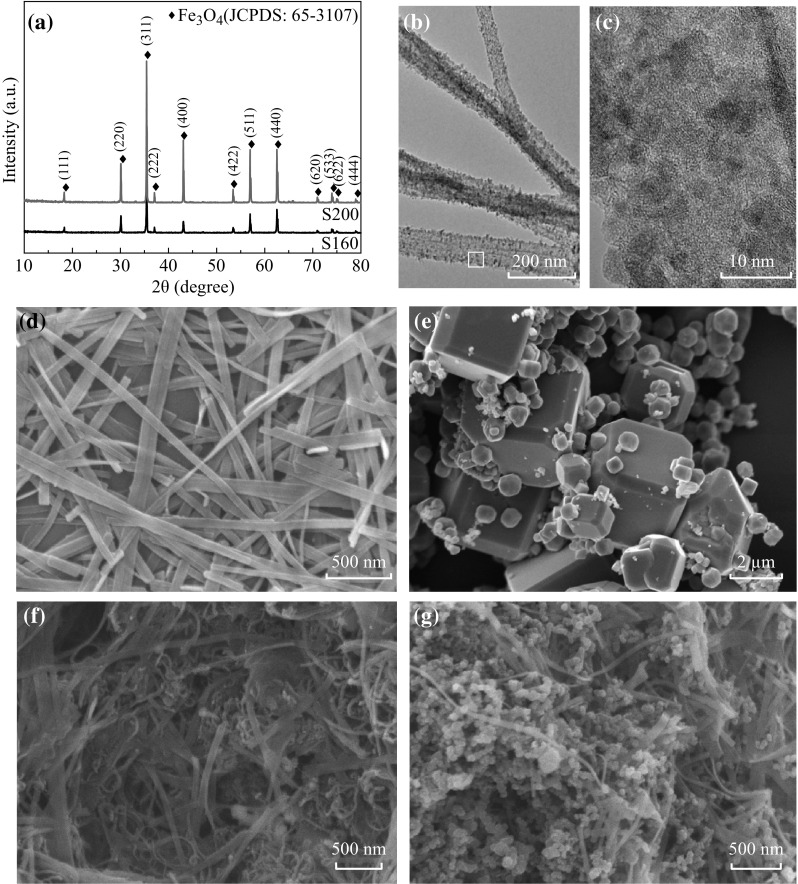
**a** XRD patterns of Fe_3_O_4_ products prepared at 160 °C for 20 h (S160) and 200 °C for 8 h (S200). **b** A typical TEM image of belt-like Fe_3_O_4_ products. **c** HRTEM image of the belt region marked with a *black pane* in panel **b**. SEM images of Fe_3_O_4_ products prepared at **d** 160 °C for 20 h and **e** 200 °C for 8 h. SEM images of belt-like Fe_3_O_4_ mixed with **f** MWNT and **g** acetylene black

The chemical composition of the as-prepared S160 and S200 products is shown in Fig. 1a, in which all the diffraction peaks of each product can be unambiguously indexed to the cubic structure of Fe_3_O_4_ (JCPDS No. 65-3107). The SEM images in Fig. 1d, e clearly show the distinct morphology difference between these two products, in which S160 and S200 are of belt-like and polyhedral morphologies, respectively. Upon closer scrutiny, most Fe_3_O_4_ belt is tens of microns long, ca. 100-nm wide and ca. 10-nm thick (Fig. 1d). In contrast, the polyhedral Fe_3_O_4_ product is less uniform in size, ranging from 1 to 4 m (Fig. 1e). Close inspection of the representative TEM image of Fe_3_O_4_ belt in Fig. 1b reveals that its surface is rough in texture, and is full of small deposited particles of similar sizes. The HRTEM image in Fig. 1c further demonstrates the relatively poor crystallinity of the belt, in which the finite-sized Fe_3_O_4_ particles of ca. 5 nm spread over the amorphous belt-matrix. Nonetheless, due to the size range of these particles, there are no obvious XRD peaks broadening in Fig. 1a. Even the belt product has relatively poor crystallinity, most of them are still crystalline.

**Fig. 2:**
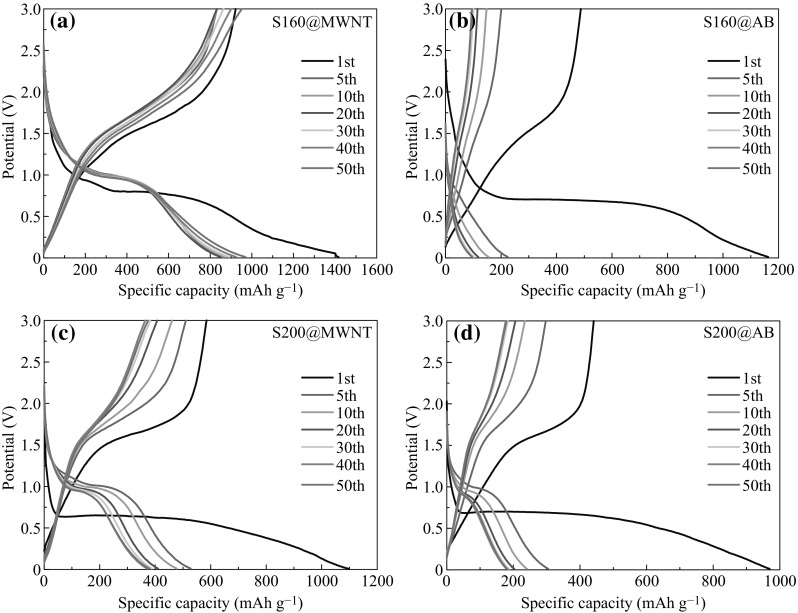
Charge-discharge voltage profiles of **a** S160@MWNT (2.04 mg)/Li, **b** S160@AB (2.22 mg)/Li, **c** S200@MWNT (2.1 mg)/Li, and **d** S200@AB (2.4 mg)/Li cells cycled between 0.01 and 3 V at 0.2 C rate

In order to study the impact of morphology of Fe_3_O_4_ and conductive agent on lithium storage properties, we utilize S160 and S200 mixed with two kinds of conductive agents (i.e., AB and MWNT) to assemble four different half-cells. Separately, the charge–discharge cycling was carried out for S160@MWNT (2.04 mg)/Li, S160@AB (2.22 mg)/Li, S200@MWNT (2.1 mg)/Li, and S200@AB (2.4 mg)/Li cells in the voltage window of 0.01–3 V at 0.2 C rate. The voltage versus capacity profiles are shown in Fig. 2. During the 1st cycle, the voltage plateau appears at ~0.7 V mainly due to the reduction of Fe^3+^ and Fe^2+^ to Fe^0^, and then the curve slopes down to the cut voltage of 0.01 V, which are typical characteristics of voltage trends for the Fe_3_O_4_ electrodes [22–26]. The subsequent discharge curves of these four cells are all different from the first ones, suggesting drastic, Li^+^ —driven, structural or textural modifications [27]. Generally, the lithium storage mechanism of metal oxides is based on a redox conversion reaction, where the metal oxides are reduced to metallic nanocrystals dispersed in Li_2_O matrix upon lithiation and then reversibly restored to their initial oxidation states during delithiation [28]. Along this line, electrochemical lithium storage in Fe_3_O_4_ follows the conversion reaction mechanism described by Eq. (1): [27, 29].

1 $${\text{Fe}}_{ 3} {\text{O}}_{ 4} + {\text{ 8Li}}^{ + } + {\text{ 8e}}^{ - }\leftrightharpoons 4 {\text{Li}}_{ 2} {\text{O}} + {\text{ 3Fe}}.$$

The formation of Li_2_O and Fe in the forward reaction is thermodynamically favorable during the discharge process. However, the extraction of Li^+^ ion from Li_2_O in the reverse process is more difficult, which suggests that a certain extent of irreversibility is inevitable. This conversion reaction provides the dominant contribution to the lithium storage capacity of Fe_3_O_4_ material and gives rise to a high initial discharge capacity of 1417, 1163, 1097, and 970 mAh g^−1^ for S160@MWNT/Li, S160@AB/Li, S200@MWNT/Li, and S200@AB/Li cells, respectively, corresponding to 12.3, 10.1, 9.5, and 8.4 mol consumption of Li per mole of Fe_3_O_4_ anode material. A reversible charge capacity of 859, 224, 528, and 305 mAh g^−1^ can be delivered after the 5th cycle, which leads to irreversible capacity loss of 39.4, 80.7, 51.9, and 68.6 % for S160@MWNT/Li, S160@AB/Li, S200@MWNT/Li, and S200@AB/Li cells, respectively. Such initial irreversible capacity loss is commonly ascribed to the formation of solid-electrolyte interface layer and some other side reactions [30].

**Fig. 3:**
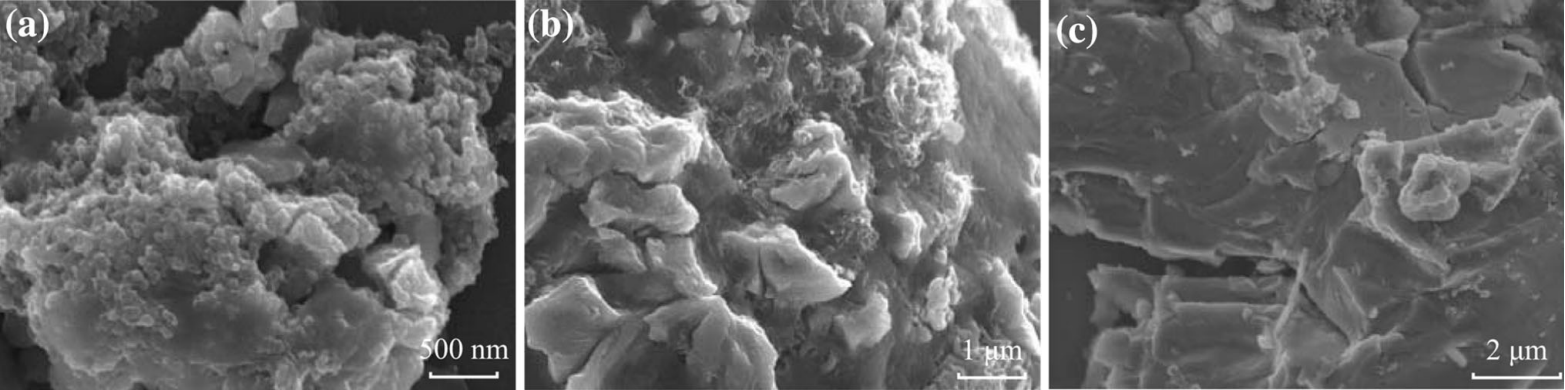
SEM images of active materials in **a** S160@AB/Li, **b** S200@MWNT/Li, and **c** S200@AB/Li cells after 50th-cycled capacity retention

**Fig. 4:**
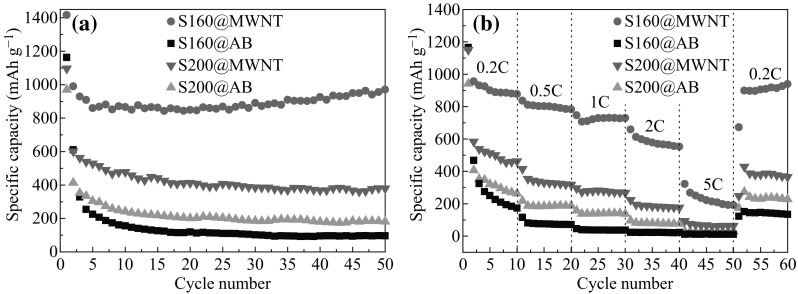
**a** Capacity retention of S160@MWNT (2.04 mg)/Li, S160@AB (2.22 mg)/Li, S200@MWNT (2.1 mg)/Li, and S200@AB (2.4 mg)/Li cells cycled between 0.01 and 3 V at 0.2 C rate. **b** Rate capabilities of S160@MWNT (1.86 mg)/Li, S160@AB (2.04 mg)/Li, S200@MWNT (2.34 mg)/Li, and S200@AB (1.92 mg)/Li cells at different rates: 0.2, 0.5, 1, 2, 5 C

The cycling performances of these cells are depicted in Fig. 4a at 0.2 C rate between 0.01 and 3.0 V. From the 5th cycle onward, S160@MWNT/Li cell exhibits the most excellent cyclic capacity retention of 970 mAh g^−1^ after 50th cycle. Following that, in order, are S200@MWNT/Li, S200@AB/Li, and S160@AB/Li cells with capacities of 380, 182, and 96 mAh g^−1^ after 50th cycle, respectively. Collectively, the capacity retention of Fe_3_O_4_ is somewhat disappointing because of the aggregation of Fe_3_O_4_ particles during cycling and large volume variation (>200 %) [27, 31]. This point can be confirmed by the SEM images of active materials in S160@AB/Li, S200@MWNT/Li, and S200@AB/Li cells after 50th-cycled capacity retention in Fig. 3. It shows that the original shape of Fe_3_O_4_ particles can hardly be distinguished due to their aggregation and volume expansion. More importantly, Fig. 4a strongly indicates that the morphologies of anode material and conductive agent have critical impact on LIBs cycling performances. It is unanimously accepted that the approaches for enhancing Li^+^ and electron transport kinetics in LIBs include designing electrode materials with high Li^+^ diffusion constants and coating the electrolytically active material with a conductive layer to improve electron transport. Moreover, it has often been taken that reductions in the characteristic dimensions of the electrolytically active material are more effective in improving LIBs cycling performance than increases in ion diffusivity *D*, because the characteristic time constant *t* for diffusion is proportional to the square of the diffusion length *L* (i.e., *t~L* ^2^/*D*). Along this line, nanoscale electrodes have exceptionally shorten Li^+^ and electron transport lengths. Therefore, the ideal electrode architecture for providing efficient Li^+^ and electron transport should consist of a three-dimensional interpenetrating nano-network of Li^+^ and electron pathways. This is the exact case for S160@MWNT/Li cell in our study. As shown in Fig. 1f, the intertwined belt-like Fe_3_O_4_ and tubular MWNT provide effective Li^+^ channel and rapid electron channel, respectively. As such, the recombination of Li^+^ and electron can be greatly suppressed, and thus the efficient separation of positive and negative charges is typically achieved, resulting in its superior cycling performance. In contrast, the capacity retention of the other three cells is much inferior, presumably because of their entangled Li^+^ and electron pathways.

To further validate the above statements, rate capabilities of S160@MWNT (1.86 mg)/Li, S160@AB (2.04 mg)/Li, S200@MWNT (2.34 mg)/Li, and S200@AB (1.92 mg)/Li cells were conducted in the voltage range of 0.01 and 3 V at different cycling rates (with the same rates for both charge and discharge). Figure 4b shows the S160@MWNT/Li cell exhibits the most superior rate capability, the next is S200@MWNT/Li cell, followed by S200@AB/Li and S160@AB/Li cells, which is in the same priority order as their cycling performances. Notably, the cyclability and rate capability of MWNT-involved cells (i.e., S160@MWNT/Li and S200@MWNT/Li cells) greatly outperform those of AB-involved cells (i.e., S160@AB/Li and S200@AB/Li cells). This is because micron-sized soft MWNT not only facilitates the rapid and effective electron transport between electrode materials and the current collector, but also tightly twists and traps the active material, so that the strain caused by the volume variation of Fe_3_O_4_ during charge–discharge cycles can be effectively alleviated. This observation strongly evidences that the morphology of conductive agent, rather than that of anode material, is of utmost importance in determining LIBs storage performances. More specifically, if the topology of electronic pathways is such that all the particles of conductive agent are effectively wired, much faster charging and discharging rates are achieved, and thus leading to superior cyclability and rate capability. Otherwise, even though the morphology of Fe_3_O_4_ anode material is conducive for Li^+^ ions to travel (e.g., S160@AB/Li cell), the electrochemical properties of such cells are inferior even at low current densities (Fig. 4).

Based on the above discussion, if the morphology of conductive agent of each cell is identical, the electrochemical performances of these cells are mainly determined by the morphology of anode material. However, the morphology of anode material plays a subordinate role on the LIBs storage performance. For instance, by using the same type of MWNT conductive agent, the cyclability and rate capability of S160@MWNT/Li cell is much superior to those of the S200@MWNT/Li cell. This is because the two-dimensional Fe_3_O_4_ belt presents a finite lateral size and enhanced open-edges, which facilitate lithium-ion and electron diffusion through active materials and better withstand the large volume change during the charge/discharge process [32, 33]. However, the electrochemical performances of S160@AB/Li and S200@AB/Li cells seem to run counter to the above trend, hinting at the presence of morphology-matching principle for the electrode materials. Under the condition of any given type of conductive agent, the contact degree of the conductive agent and anode material actually determines the LIBs electrochemical performances. In comparison to polyhedral Fe_3_O_4_ particles, the rigid Fe_3_O_4_ belts cannot integrate AB particulates well (Fig. 1g) and hence resulting in the worst cyclability and rate capability (Fig. 4).

**Fig. 5:**
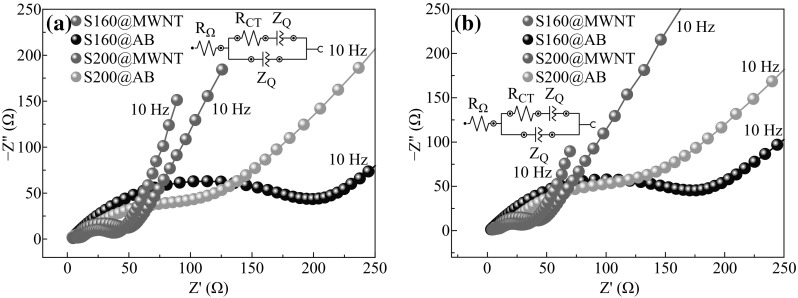
Electrochemical impedance spectroscopy of the cells after **a** 50th-cycled capacity retention test and **b** 60th-cycled rate capability test. *Insets* equivalent circuits used to fit the impedance data

To further validate the above analysis, EIS measurements of S160@MWNT/Li, S160@AB/Li, S200@MWNT/Li, and S200@AB/Li cells after 50th-cycled capacity retention test (Fig. 5a) and 60th-cycled rate capability test (Fig. 5b) were conducted to examine the kinetics of lithium-ion transfer by using a CHI660D electrochemical workstation. The impedance of the electrochemical system was interpreted in terms of Nyquist plots, which describe the gain and phase of the frequency response in polar coordinates. The Nyquist plots for these four cells in two cases are shown in Fig. 5, which were acquired individually under their open-circuit voltage state. Each plot consists of a semicircle in the high-frequency region and a sloping line in the low frequency, which was attributed to the charge transfer process and the mass transfer of lithium ions, respectively. The electrochemical system can be simply fitted by equivalent circuits in the insets of Fig. 5, where *R*_Ω_ is the ohmic resistance, *R*_CT_ is the charge transfer resistance, *Z*_Q_ is the constant phase element. The fitting results are summarized in Table 1. At very high frequencies (above 10 kHz), only the ohmic resistance can be observed, which is mainly due to external connections, contact resistance, and ionic conduction within the electrolyte [18]. Because of the same electrolyte and identical cell configurations, all electrodes have a similar value of *R*_Ω_ for the ohmic resistance (below 7 Ω) obtained from the fitting results, and this can be easily ascertained from the high-frequency intercept with the real axis (Z axis) in the Nyquist plots. A semicircle appears in the frequency region from 10 Hz to 10 kHz for each cell, which can be attributed to an interfacial charge transfer. The charge transfer resistance can also be determined by fitting the equivalent circuits or directly measuring the diameter of the semicircle in the Nyquist plots. From Table 1, the order from small to large *R*_CT_ values is S160@MWNT/Li, S200@MWNT/Li, S200@AB/Li, and S160@AB/Li cells, indicating that the electron transfer would be much easier with the presence of tubular conductive agent. Thereby, based on the EIS measurements, the order from superior to inferior electrochemical performances of these cells is predicted as S160@MWNT/Li, S200@MWNT/Li, S200@AB/Li, and S160@AB/Li cells, which is in excellent consistency with the conclusion deduced from Fig. 5.

**Table 1:** Electrochemical impedance spectroscopy fitting results for cells after 50th-cycled capacity retention and 60th-cycled rate capability test

Resistance (Ω)		S160@MWNT	S160@AB	S200@MWNT	S200@AB
After 50th-cycled	*R*_Ω_	6.48	3.83	3.73	2.74
Capacity retention test	*R*_CT_	27.1	214	44.7	122
After 60th-cycled rate	*R*_Ω_	1.41	2.63	2.69	2.75
Capability test	*R*_CT_	22.3	197	37.8	146

## Conclusions

In summary, we utilize granular and belt-like Fe_3_O_4_ anode materials and two types of conductive agents (including AB and MWNT) as prototype systems to investigate their morphology impact on lithium storage performances. We find that the morphology of conductive agent plays a decisive role on electrochemical performances. After 50th cycle, the capacity of MWNT-involved cells (i.e., 970 mAh g^−1^ for S160@MWNT/Li cell and 380 mAh g^−1^ for S200@MWNT/Li cell) is much higher than that of the AB-involved cells (i.e., 182 and 96 mAh g^−1^ for S200@AB/Li and S160@AB/Li cells, respectively). Provided that the morphology of conductive agent of each cell is identical, the electrochemical performances of these cells are mainly determined by the morphology of anode material as well as the contact degree of these electrode materials.
